# Immune Biomarkers in Metastatic Castration-resistant Prostate Cancer

**DOI:** 10.1016/j.euo.2022.04.004

**Published:** 2022-04-28

**Authors:** María Dolores Fenor de la Maza, Khobe Chandran, Jan Rekowski, Irene M. Shui, Bora Gurel, Emily Cross, Suzanne Carreira, Wei Yuan, Daniel Westaby, Susana Miranda, Ana Ferreira, George Seed, Mateus Crespo, Ines Figueiredo, Claudia Bertan, Veronica Gil, Ruth Riisnaes, Adam Sharp, Daniel Nava Rodrigues, Pasquale Rescigno, Nina Tunariu, Xiao Qiao Liu, Razvan Cristescu, Charles Schloss, Christina Yap, Johann S. de Bono

**Affiliations:** a The Institute of Cancer Research, London, UK; b The Royal Marsden Hospital, London, UK; c Merck & Co., Inc., Kenilworth, NJ, USA; d Royal Free Hospital, London, UK; e Candiolo Cancer Institute, FPO- IRCCS, Candiolo, Turin, Italy

**Keywords:** PD-L1, T-cell–inflamed gene expression, profile, SOX2, Correlation, Overall survival

## Abstract

**Background:**

Metastatic castration-resistant prostate cancer (mCRPC) is a heterogeneous disease in which molecular stratification is needed to improve clinical outcomes. The identification of predictive biomarkers can have a major impact on the care of these patients, but the availability of metastatic tissue samples for research in this setting is limited.

**Objective:**

To study the prevalence of immune biomarkers of potential clinical utility to immunotherapy in mCRPC and to determine their association with overall survival (OS).

**Design, setting, and participants:**

From 100 patients, mCRPC biopsies were assayed by whole exome sequencing, targeted next-generation sequencing, RNA sequencing, tumor mutational burden, T-cell–inflamed gene expression profile (TcellinfGEP) score (Nanostring), and immunohistochemistry for programmed cell death 1 ligand 1 (PD-L1), ataxia-telangiectasia mutated (ATM), phosphatase and tensin homolog (PTEN), SRY homology box 2 (SOX2), and the presence of neuroendocrine features.

**Outcome measurements and statistical analysis:**

The phi coefficient determined correlations between biomarkers of interest. OS was assessed using Kaplan-Meier curves and adjusted hazard ratios (aHRs) from Cox regression.

**Results and limitations:**

PD-L1 and SOX2 protein expression was detected by immunohistochemistry (combined positive score ≥1 and >5% cells, respectively) in 24 (33%) and 27 (27%) mCRPC biopsies, respectively; 23 (26%) mCRPC biopsies had high TcellinfGEP scores (>–0.318). PD-L1 protein expression and TcellinfGEP scores were positively correlated (phi 0.63 [0.45; 0.76]). PD-L1 protein expression (aHR: 1.90 [1.05; 3.45]), high TcellinfGEP score (aHR: 1.86 [1.04; 3.31]), and SOX2 expression (aHR: 2.09 [1.20; 3.64]) were associated with worse OS.

**Conclusions:**

PD-L1, TcellinfGEP score, and SOX2 are prognostic of outcome from the mCRPC setting. If validated, predictive biomarker studies incorporating survival endpoints need to take these findings into consideration.

## 1 Introduction

Prostate cancer is the second most prevalent cancer in men worldwide and is estimated to result in the death of >70 000 men in Europe in 2020 [1,2]. Although most advanced prostate cancers initially respond to androgen deprivation therapy, second-generation androgen inhibitors, taxane-based chemotherapies, and radionuclide therapies, advanced disease remains fatal [3].

Recent phase 2 and phase 3 clinical trials have shown that tumor molecular characterization could further improve metastatic castration-resistant prostate cancer (mCRPC) outcomes through disease stratification [4,5]. However, the availability of mCRPC biopsies for analysis is scant. Durable responses to immune checkpoint inhibitors including antiprogrammed cell death 1 ligand 1 (anti-PD-1/PD-L1) agents have also been observed in a small subset of patients, indicating that patient selection is critical to optimizing benefit in mCRPC [6,7].

In this study, we examined immune biomarkers in mCRPC, analyzing 100 fresh mCRPC biopsies for PD-L1 protein expression, T-cell–inflamed gene expression profile (TcellinfGEP) mRNA scores, SRY homology box 2 (SOX2), and other biomarkers of interest. We determined the prevalence of these biomarkers using next-generation sequencing, targeted and whole exome sequencing, RNA sequencing, and immunohistochemistry (IHC) to test their associations with clinical characteristics and overall survival (OS).

## 2 Patients and methods

### 2.1 Patients

The study population included 100 patients with mCRPC who had prospectively had a fresh mCRPC biopsy at the Royal Marsden Hospital (RMH; London, UK) between October 2014 and July 2019. The date of biopsy was considered the *index date* for the study analyses. Patient selection was done according to the inclusion criteria for the study, prespecified in the study protocol. All patients were 18 yr of age or older, and had histologically or cytologically confirmed metastatic adenocarcinoma of the prostate without small cell histology. All patients were treated with at least one second-generation antiandrogen therapy and at least one regimen of chemotherapy that contained docetaxel in the mCRPC or metastatic hormone-sensitive prostate cancer setting. In addition to biomarker data, demographics, clinical characteristics, and outcome data for each patient were extracted from patient records.

### 2.2 Specimen characteristics

Archived tissue samples from all identified patients were retrieved, and slides (including one hematoxylin and eosin slide) were cut for biomarker testing. All mCRPC biopsies were prospectively acquired using an approved protocol for prostate cancer molecular characterization at the RMH (04/Q0801/60). All patients provided written informed consent.

### 2.3 Assay methods for biomarkers of interest

The assays conducted in this study are presented in Supplementary Tables 1 and 2, but have largely been described previously [8–16].

### 2.4 Study design

This was a single-center cohort study. The study was performed at the RMH, and an analysis was carried out at the Institute of Cancer Research. A retrospective approach was taken to collect demographic and clinical data as well as clinical outcomes from electronic medical records. The collected data span the period from October 2014 to July 2019, whereas data collection started in August 2019 and was completed in June 2020. The last day of clinical follow-up was June 2, 2020.

### 2.5 Sample size and power calculations

The sample size of 100 patients was based on patient availability rather than on formal power calculations, as the focus of this observational study was on estimation and precision instead of hypothesis testing. Simulation using the statistical software R (version 4.0.0 R Foundation for Statistical Computing, Vienna, Austria), however, yielded that given a 40% PD-L1 positivity, 100 samples allowed for 79.4% statistical power to detect a hazard ratio (HR) of 0.52 in favor of PD-L1–positive patients. This assumed an exponential distribution for survival in both groups, median OS of 4 mo for the entire population, 6-mo median OS among PD-L1–positive patients, a censoring rate of approximately 16% after 2 yr, and the use of a two-sided log-rank test with significance level 5%. The increased OS was an assumption made for the power calculation. If we assumed decreased OS for PD-L1 cases, we would have a similar power (82%) to detect an HR of 1.92.

### 2.6 Statistical analysis

Patient characteristics are reported as median plus first and third quartiles, or as absolute and relative frequencies depending on their level of measurement. The prevalence of each biomarker is reported with its 95% confidence interval (CI). The association between two binary biomarkers was assessed by the phi coefficient. Venn diagrams illustrate expression of biomarkers in combination with each other.

The median follow-up from the index date was calculated using the reverse Kaplan-Meier (KM) method. OS was defined as the time from the date of mCRPC biopsy (index date) to death from any cause. Patients without documented death at the time of the last follow-up were censored at the date last seen. KM plots investigated the association between each biomarker and OS. Cox regression provided adjusted hazard ratios (aHRs) with corresponding 95% CIs controlling for potential confounders and known prognostic factors (at diagnosis: Eastern Cooperative Oncology Group and Gleason score >7; at the index date: age, logtransformed prostate-specific antigen, and presence of liver metastasis). Six patients had not been treated with docetaxel before the index date for clinical reasons but were included in the final analysis. No measures were taken to impute missing data; complete case analysis was used instead. Statistical analysis was performed using R version 4.0.0.

## 3 Results

### 3.1 Population characteristics, biomarker prevalence, and correlations

A total of 100 patients were included in the analysis; the median follow-up from the index date was 56 mo; 99 patients have died. The median age at the index date was 68 yr. The median between mCRPC diagnosis and index date was 25.5 mo, with Q1 being estimated as 15.75 mo and Q3 as 36.1 mo. Of the 100 mCRPC biopsy sites, 54 (54%) were nodal, 29 (29%) bone, nine (9%) soft tissue, seven (7%) visceral, and one (1%) other; 46/84 (55%) patients had metastatic prostate cancer at diagnosis, and 24/100 (24%) patients had meta-static liver disease at the time of mCRPC biopsy. The median number of treatments was 5 (4, 6), and the median number of treatments before the index date was 3 (2, 4). Sixty patients did not have any chemotherapy line after the index date of biopsy, while 34 patients had one line of chemotherapy and six had two lines of chemotherapy. Cabazitaxel was given to 26 of these patients, docetaxel to five patients, and carboplatin-based chemotherapy to 15 patients. When looking at PD-L1, TcellinfGEP, and SOX2, there were no remarkable imbalance in treatment with cabazitaxel after biopsy (risk ratio 0.93 [95% CI: 0.37–2.36], 0.86 [0.39–1.89], and 0.63 [0.27–1.51], respectively). A total of 19 cases were classified as visually neuroendocrine (NE; ≥1% of tumor cells had NE features). Employing a cutoff of 20% of tumor cells having NE features yielded a total of nine cases with a significant number of NE tumors in the biopsy sample. Clinical characteristics by selected biomarkers are presented in Supplementary Table 3.

**Fig. 1:**
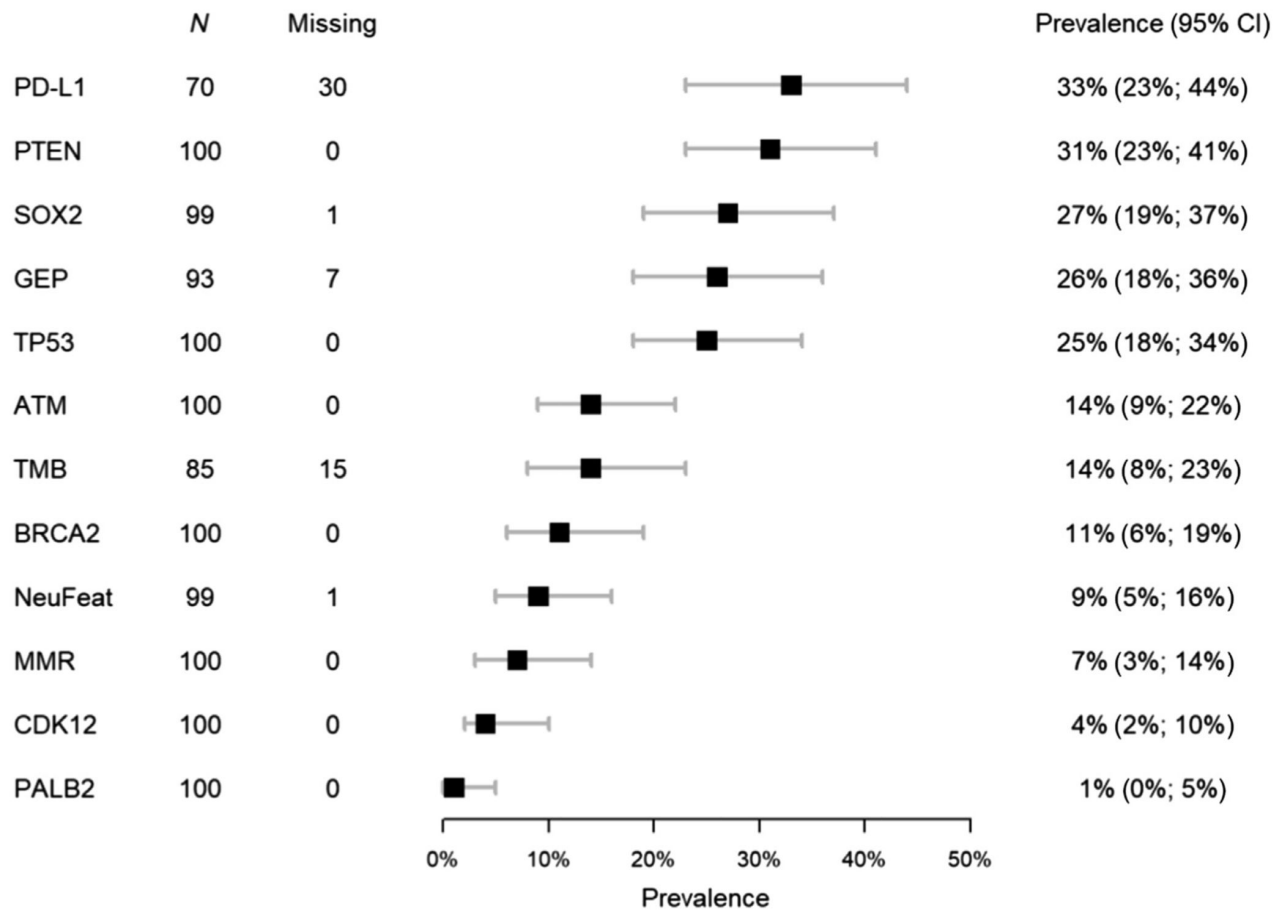
Forest plot of the prevalence of biomarkers of interest. Number of available samples (*N*), number of missing values, and the prevalence of the biomarkers of interest, calculated as the number of patients with a biomarker expressed divided by the number of patients with available data for this biomarker, are shown. A 95% confidence interval (CI) for the prevalence is given.

PD-L1 protein expression, TcellinfGEP score, and tumor mutational burden (TMB) results were available for 70, 93, and 85 samples, respectively. PD-L1 was expressed in 23/70 (33%) mCRPC biopsies; 24/93 (26%) had high Tcellinf-GEP scores and 27/99 (27%) had SOX2 IHC expression (Fig. 1). Fig. 1 depicts the prevalence of the other evaluated biomarkers.

**Fig. 2:**
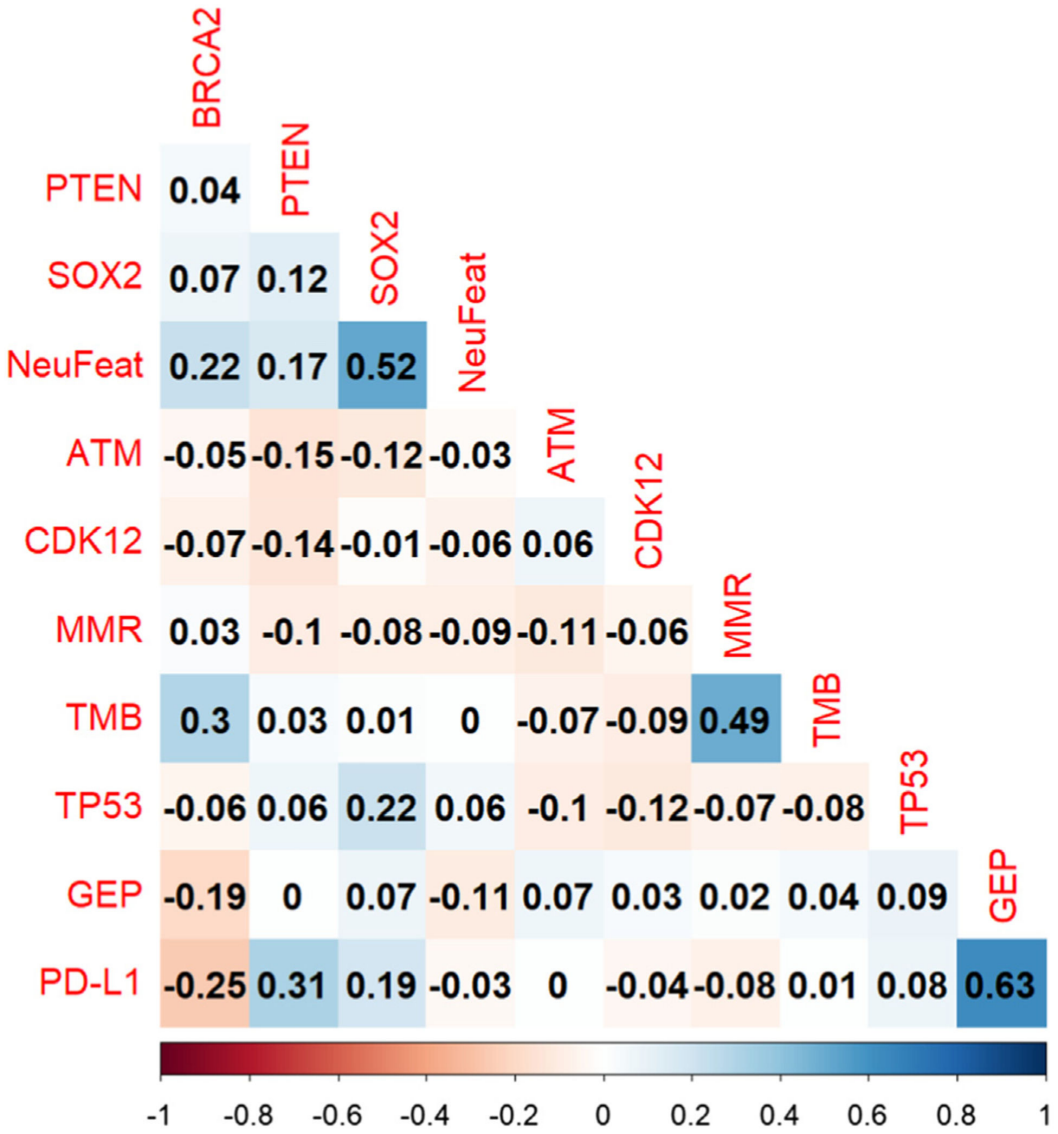
Phi coefficient to assess the correlation between the expression of different biomarkers. Strong positive correlation is represented by dark blue squares, and strong negative correlation is represented by dark red squares.

**Fig. 3:**
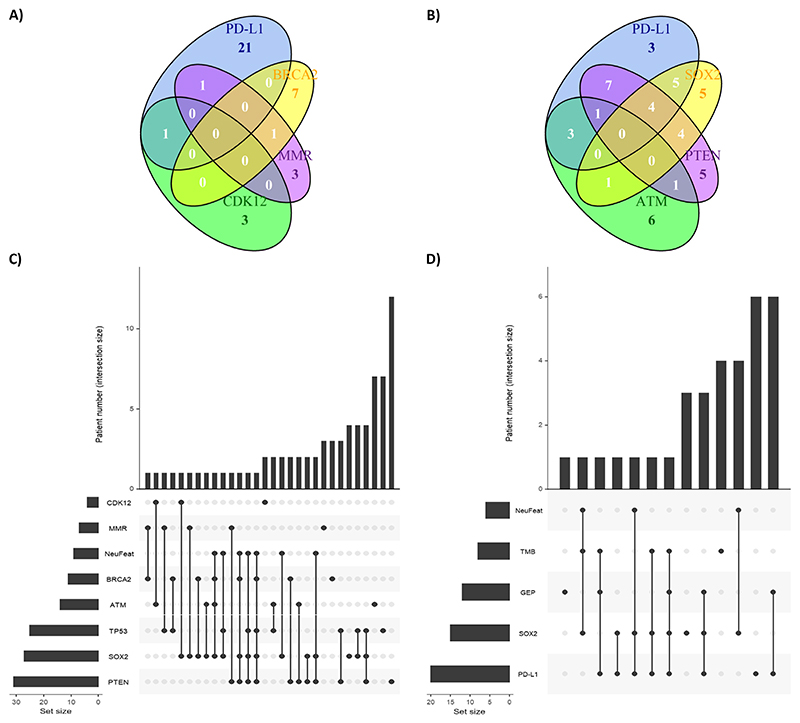
(A) Venn diagram representing the overlap of PD-L1 expression, BRCA2/CDK12 deleterious genomic alterations, and MMR deleterious genomic alterations and/or loss of protein expression. Absolute numbers of biomarker expression and coexpression are shown. The analysis is restricted to patients with available data for all four biomarkers (*N* = 70). (B) Venn diagram representing the overlap of PD-L1 expression, ATM loss of protein expression, SOX2 expression, and PTEN loss of protein expression. Absolute numbers of biomarker expression and coexpression are shown. The analysis is restricted to patients with available data for all four biomarkers (*N* = 70). (C) UpSet plot representing the most common combinations and the frequency of deleterious alterations for CDK12, BRCA2, p53, dMMR, neuroendocrine histology features, SOX2 protein expression, and ATM/PTEN loss of protein expression. Horizontal bars in the bottom left show absolute frequencies of biomarkers one by one. Vertical bars on the top show absolute frequencies for biomarker combinations (as indicated by filled dots). Twenty-four biopsies did not have any of the eight biomarkers (*N* = 99). (D) UpSet plot representing the most common combinations and the frequency of isolated alterations for high TMB, high TcellinfGEP score >–0.318, and PD-L1– and SOX2-positive protein expression. Horizontal bars in the bottom left depict the absolute frequencies of biomarkers one by one. Vertical bars on the top show absolute frequencies for biomarker combinations (as indicated by filled dots). Twenty-three biopsies did not have any of the five biomarkers (*N* = 56). ATM = ataxia-telangiectasia mutated; BRC2 = breast cancer 2; CDK12 = cyclin-dependent kinases 12; dMMR = DNA mismatch repair; PD-L1 = programmed cell death 1 ligand 1; PTEN = phosphatase and tensin homolog; SOX2 = SRY homology box 2; TcellinfGEP = T-cell–inflamed gene expression profile; TMB = tumor mutational burden.

PD-L1 expression and high TcellinfGEP score were positively correlated (phi 0.63 [0.45; 0.76]). The percentages of biopsy sites by PD-L1 and TcellinfGEP are presented in Supplementary Table 4. There was a positive correlation between SOX2 IHC expression and NE features in the histology (phi 0.52 [0.36; 0.65]). No other biomarkers had strong correlations; however, TMB and DNA mismatch repair (dMMR) were moderately correlated (phi 0.49 [0.31; 0.64]), and 71% of dMMR samples had high TMB (Fig. 2 and Supplementary Table 5). Coexpression of selected biomarkers is presented in Fig. 3A and B. Among the five samples with MMR loss for which PD-L1 was available, one (20%) had detectable PD-L1 ≥1; one of the three cyclin-dependent kinases 12 (CDK12) altered samples also had detectable PD-L1 ≥1 (33%), but none (0%) of the six breast cancer 2 (BRCA2) mutated mCRPC cases expressed PD-L1 (Fig. 3A). Of 14 samples with phosphatase and tensin homolog (PTEN) loss for which PD-L1 was available, eight (57%) had PD-L1 ≥1; four of the 12 ataxia-telangiectasia mutated (ATM) IHC loss samples had PD-L1 ≥1 (33%), and nine of the 19 mCRPC cases that expressed SOX2 showed PD-L1 expression (47%).

The most common biomarker combinations and the frequency of isolated alterations are represented in Fig. 3C and D. Fig. 3C illustrates the frequency of co-occurrence among CDK12, dMMR, BRCA2, ATM, TP53, SOX2, NE histology features, and PTEN (*n* = 99). Twenty-four patients did not have any of these alterations, with conversely only one patient having the maximum of five detected positive results (BRCA2 and TP53 alterations, SOX2 high expression, NE histology features, and PTEN loss). An UpSet plot for PD-L1, TcellinfGEP, TMB, NE histology features, and SOX2 restricted to patients with available biomarker data (*n* = 56) reveals that in seven/15 (47%) patients with SOX2 expression, none of TcellinfGEP, PD-L1, or TMB was high (Fig. 3D).

### 3.2 dMMR and TMB characterization

Overall, seven biopsies had dMMR; five of these had deleterious genomic alterations, and two had loss of MSH6 and MSH2 proteins without detectable deleterious genomic alterations (71% and 29%, respectively). Of the five samples with deleterious genomic alterations, three (60%) had germline alterations, three (60%) had biallelic hits, and three (60%) had concomitant MMR protein loss by IHC, suggesting that MMR protein deleterious mutation does not always result in IHC loss of expression (Supplementary Table 5). Patient 039 had mCRPC with biallelic alteration with a mutation of uncertain significance in MSH6 (S241G); this sample had MMR protein loss by IHC. Five (71%) of these seven samples with dMMR had high TMB (Supplementary Table 5). Patients with high TMB had worse OS (aHR 1.58 [CI: 0.79; 3.16]; Supplementary Fig. 1B).

### 3.3 Correlation between biomarkers of interest and survival outcomes

**Fig. 4:**
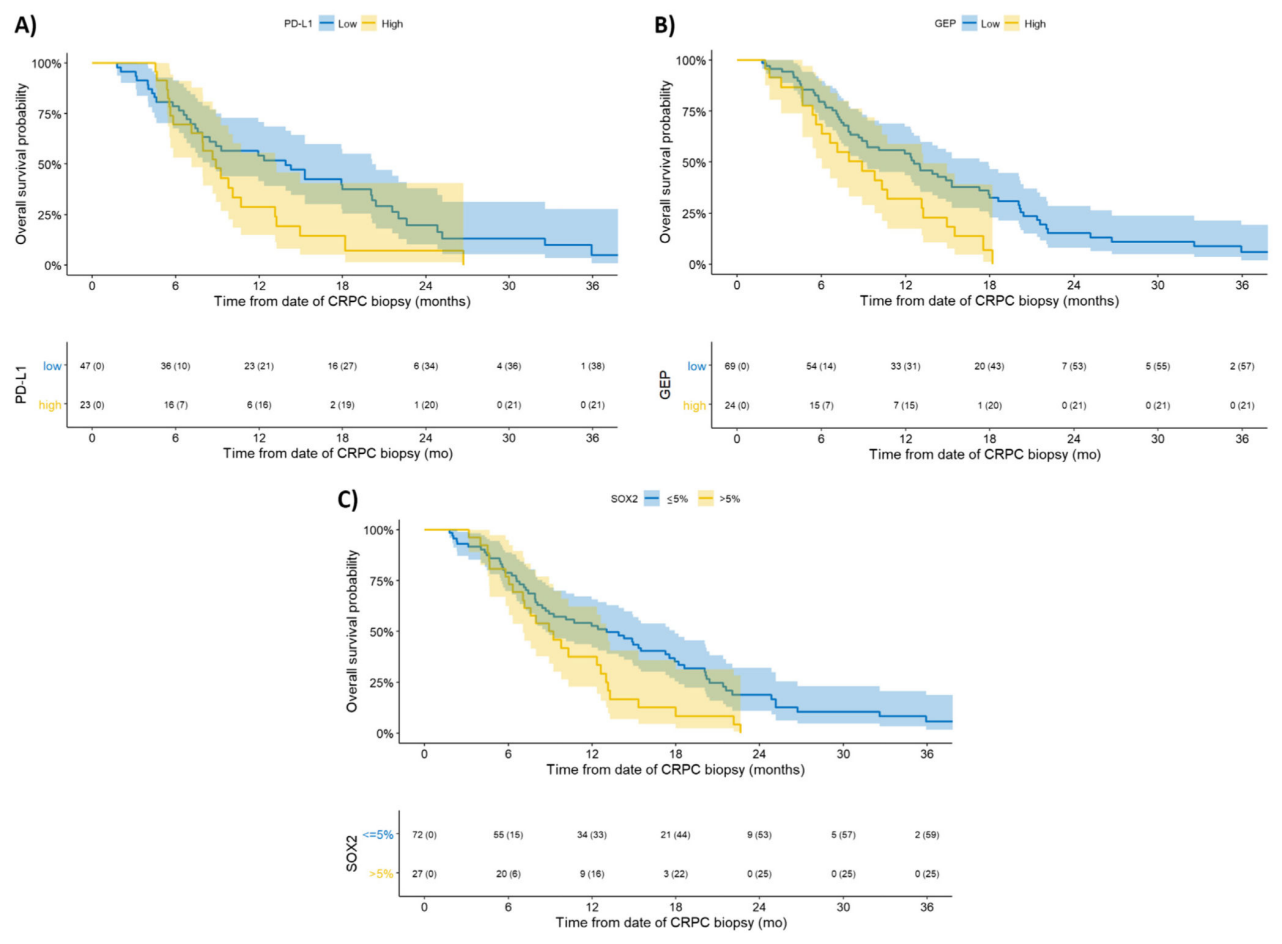
Kaplan-Meier plots showing overall survival (OS) by biomarker of interest for low versus high PD-L1 protein expression. (A) High PD-L1 expression defined as a combined positive score (CPS) of ≥1 versus low PD-L1 expression. The median OS was shorter for patients with high PD-L1 expression (9 mo) than for patients with low PD-L1 expression (14 mo). (B) A high TcellinfGEP score, defined as a TcellinfGEP score of >–0.318, versus a low TcellinfGEP score. The median OS was shorter for patients with a high TcellinfGEP score (9 mo) than for patients with a low TcellinfGEP score (13 mo). (C) High SOX2 expression, defined as a percentage of cells with SOX2 expression of >5%, versus low SOX2 expression. The median OS was shorter for patients with high SOX2 expression (9 mo) than for patients with low SOX2 expression (13 mo). Note that survival curves have been truncated at 36 mo due to sparse data. CRPC = castration-resistant prostate cancer; GEP = gene expression profile; PD-L1 = programmed cell death 1 ligand 1; SOX2 = SRY homology box 2; TcellinfGEP = T-cell–inflamed gene expression profile.

**Fig. 5:**
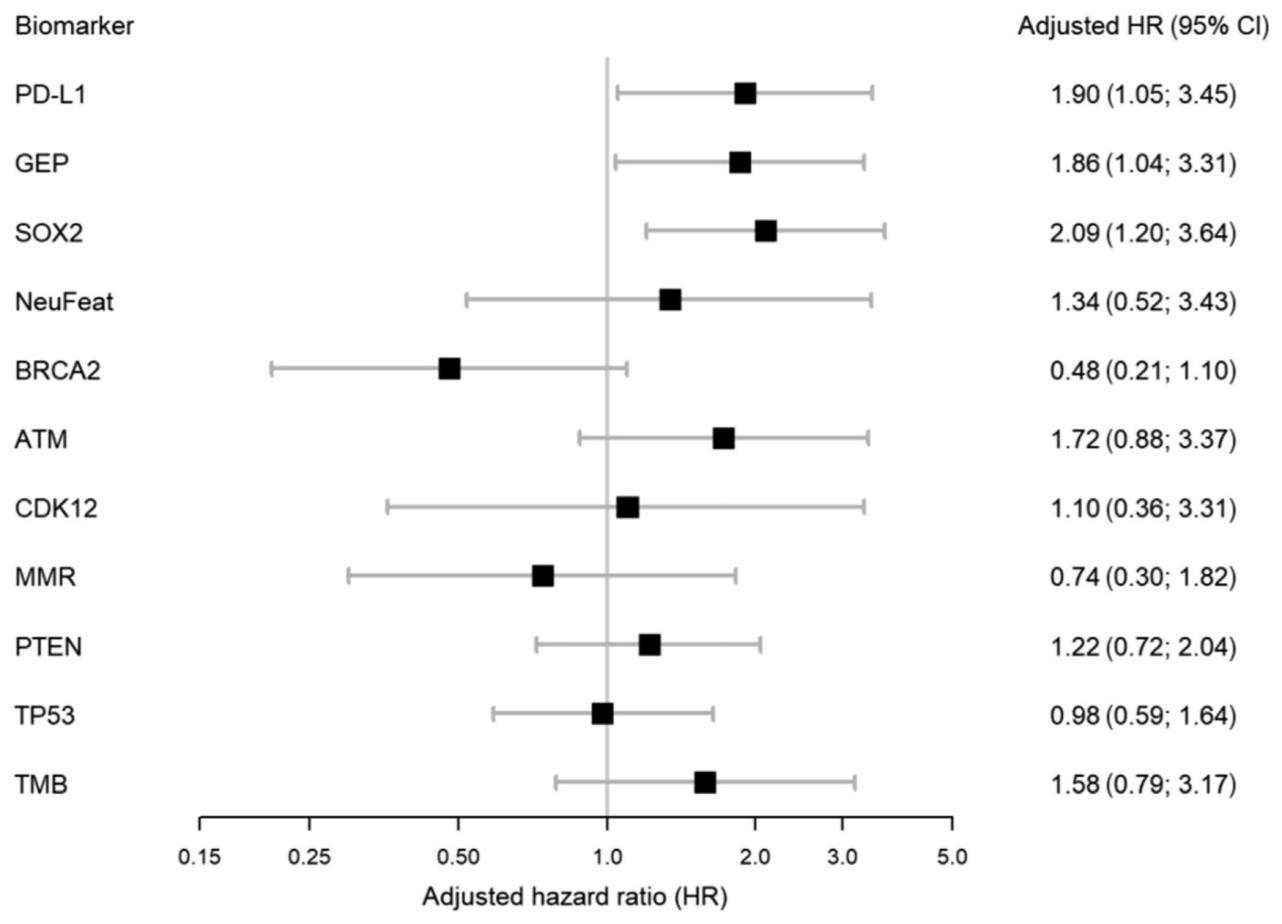
Forest plot of adjusted hazard ratios for biomarkers of interest. Hazard ratios (HRs) and their respective 95% confidence intervals (CIs) are from a Cox regression model with the respective biomarker of interest as explanatory variable and controlling for diagnostic ECOG and diagnostic Gleason score >7 as wells as age, log-transformed PSA, and presence of liver metastasis (all at the index date). ECOG = Eastern Cooperative Oncology Group; PSA = prostate-specific antigen.

KM plots revealed worse OS for patients whose mCRPC samples were PD-L1 positive or SOX2 positive, or who had high TcellinfGEP (Fig. 4). No other biomarkers showed an apparent association with OS (Supplementary Fig. 1). Adjusted HRs and 95% CIs from multivariable Cox regression yielded consistent findings (Fig. 5): PD-L1–positive expression was associated with an aHR of 1.90 (1.04; 3.45), high TcellinfGEP score with an aHR of 1.86 (1.04; 3.31), and SOX2-positive expression with an aHR of 2.09 (1.20; 3.63). Although the precision of the estimate was lower due to more unbalanced groups, Cox regression also indicated worse prognosis for patients with ATM loss (aHR 1.72 [0.88; 3.37]) and those with high TMB (aHR: 1.58 [0.79; 3.17]), and improved OS for patients with BRCA2 deleterious alterations (aHR: 0.48 [0.21; 1.10]). Of note, of the 11 patients with BRCA2 deleterious alterations, eight were treated with olaparib and/or carboplatin.

### 3.4 mRNA signatures

Having higher values of mRNA signatures representing glycolysis and proliferation, gMDSC and mMDSC pathways were associated with worse OS with aHRs (per increase in standard deviation) of 1.50 (1.10; 2.00) and 2.00 (1.40; 2.70), and 1.30 (1.00; 1.70) and 1.30 (1.00; 1.70), respectively (see Supplementary Fig. 2 for aHR for OS by mRNA signature). Supplementary Fig. 3 indicates no notable correlations between the mRNA signatures and DNA features measured in the study.

## 4 Discussion

In this study, we report that PD-L1, SOX2 IHC expression, and high TcellinfGEP scores are detected in 33%, 27%, and 26% of mCRPC biopsies, respectively, and are associated with shorter OS. To our knowledge, this is the first study reporting that these biomarkers, when studied in the mCRPC setting, are associated with OS. We also show that PD-L1 expression and high TcellinfGEP score are correlated positively; prospective studies should validate these findings, which are relevant to their study as putative predictive biomarkers in phase 3 trials.

Our study has inherent limitations related to its retrospective, single-center design, including the heterogeneity of treatment regimens administered to the patients and the availability and completeness of treatment response data. However, all patients received at least one novel hormonal agent, and 94/100 patients received docetaxel. While the sample size was relatively small, strengths of our study include the deep and novel analyses of mCRPC biopsy specimens including a wide range of biomarkers relevant to immune therapy, and the ability to assess OS on all patients. These findings would benefit from replication in other mCRPC cohorts. Our study population included patients whose disease had already progressed to the most effective therapies. This could limit the generalizability of our findings.

Although previous research has suggested that PD-L1 expression may be a biomarker predictive of prognosis in prostate cancer, these reports mainly assayed primary tumors, while our study analyzed mCRPC biopsies. Two studies showed that PD-L1 expression could be an independent indicator of biochemical recurrence [17,18], whereas another study reported that PD-L1 expression is associated with a higher risk of clinical progression in men with node-positive prostate cancer [19]. A limitation of such data published based on localized disease from primary prostate tissue is that biomarkers may change as cancers progress to the metastatic setting. In addition, these studies did not assess the association between PD-L1 and OS in the metastatic setting.

SOX2 is associated with lineage plasticity, with its levels increasing in CRPC with NE-like or basal disease emergence [20]. Of note, the lack of consensus to define NE prostate cancer could explain discrepancies between SOX2 expression and NE features assessed by histology. In our cohort, SOX2 IHC expression and NE histological features were correlated, and 47% of the SOX2-positive samples coexpressed PD-L1. Based on our results, future studies should test how NE changes impacts PD-L1 upregulation and sensitivity to PD-1/PD-L1 blockade in mCRPC. Prospective studies should also address how these observations tie in with the evolution of NE prostate cancer and provide additional data regarding how these markers change as the disease transitions following treatment failures.

TMB is another emerging biomarker reported to predict response to PD-1/PD-L1 targeting in a variety of tumors, and pembrolizumab is approved by the Food and Drug Administration for TMB-high solid tumors [8,21]. Prostate cancer is known to have lower TMB than many other solid tumors [22], and the findings from this cohort in which 14% of tumor samples had high TMB are consistent with previous studies. Patients whose samples showed high TMB tended to have worse OS, although 95% CIs included the possibility of longer OS in some of these patients (aHR 1.58 [CI: 0.79; 3.16]).

Defects in DDR proteins can induce genomic instability and trigger responses to PD-1/PD-L1 targeting by producing tumor-associated neoantigens [23]. Deleterious *DDR* gene alterations have recently been reported to be associated with improved clinical outcomes in patients with bladder and renal cancer when treated with immune checkpoint inhibitors, but this strategy is still under study in mCRPC [23,24]. In our cohort, 43% and 8% of the samples with BRCA2 deleterious genetic alterations and ATM loss, respectively, had high TMB, but none of these biomarkers was associated with PD-L1 expression. Cox regression suggested worse prognosis from ATM loss tumors, unlike in previous reports [25]; however, the precision of our estimate was lower due to unbalanced groups, so this finding needs validation in prospective studies.

Exploratory analyses also found that patients with higher mRNA signatures representing glycolysis, proliferation, and gMDSC and mMDSC pathways were associated with worse OS, in keeping with the known biological implications of increased glycolysis, proliferation, and MDSC infiltration into prostate cancers [26–28]. However, little has previously been reported on these mRNA signatures specific to outcomes from metastatic prostate cancer.

## 5 Conclusions

PD-L1 expression, high TcellinfGEP scores, and SOX2 expression are associated with poorer prognosis in mCRPC.

These biomarkers could be predictive of a T-cell–inflamed tumor microenvironment in mCRPC, which merits further validation in prospective studies.

## Supplementary Material

Supplementary Material

Supplementary Appendix

## Patient summary

This study presents an analysis of immune biomarkers in biopsies from patients with metastatic prostate cancer. We describe tumor alterations that predict prognosis that can impact future studies.
