# Supplementary material for: Immune Biomarkers in Metastatic Castration-resistant Prostate Cancer
Source: Eur Urol Oncol. Author manuscript; Available in PMC 2025 Aug 8. (PMC7617991; doi:10.1016/j.euo.2022.04.004)

Supplementary Table 1. Assay methods for biomarkers of interest

**PD-L1**	Tumor PD-L1 expression was measured by IHC on a Dako Autostainer Link 48 system (Agilent Technologies) using the Dako PD-L1 IHC 22C3 pharmDx kit (Agilent Technologies) with the EnVision FLEX visualization system. A negative control was included for each subject and stain. PD-L1 positivity was defined as Combined Positive Score (CPS) ≥1, where CPS is the number of PD-L1 staining cells (tumor cells, lymphocytes, and macrophages) divided by the total number of viable tumor cells multiplied by 100. A qualified pathologist performed cell counts and CPS categorization at Neogenomics.
**TcellinfGEP score**	The TcellinfGEP was previously derived across several solid tumors (8). Tumor RNA extracted from formalin-fixed paraffin-embedded slides were analyzed on the NanoString nCounter system (Seattle, WA), and TcellinfGEP score was calculated as a weighted sum of normalized expression values for the 18 genes, as described previously (8). Messenger RNA was extracted at Almac and the NanoString assay was done at MSD. The cutoff for samples to be considered to have a high TcellinfGEP score was >-0.318.
**ATM, PTEN, SOX2, neuroendocrine histology features and dMMR**	Immunohistochemistry was conducted at the ICR and performed on 4µm-thick FFPE tissue sections with antibodies against ATM (clone Y170), MLH1 (clone ES05), PMS2 (clone EP51), MSH2 (clone FE11), MSH6 (clone EP49), PTEN (clone 138G6) and SOX2 (clone D6D9). Assays are detailed in *Supplementary Table 1* and all cases were

Confidential

	assessed by pathologists specialized in prostate cancer (DNR and BG). For ATM, PTEN and SOX2 assessment, nuclear and/or cytoplasmic protein expression was evaluated using histo-score (h-score) (9-10). ATM and PTEN loss were defined as samples with h-score <10. SOX2 expression was considered positive when the percentage of cells with SOX2 expression was higher than 5%. Presence of neuroendocrine features was determined according to visual morphology. For visual determination of a probable neuroendocrine (NE) morphology on H&E sections, we used the morphologic classification for prostate cancer with NE differentiation by Epstein et al (11).<br><br>dMMR was defined as loss of protein detected by IHC and/or a pathogenic genetic alteration in PSM2, MSH2, MSH6 or MLH1 detected by NGS associated with features of dMMR including high mutation load. Expression of MLH1, PMS2, MSH2 and MSH6 by IHC was assessed by segregating cases in a binary fashion; cases with positive nuclear staining were considered to have protein present and cases with no nuclear staining were regarded as having absent protein expression (12).
**Targeted NGS and WES**	Targeted NGS was performed as described previously (13) using a custom Generead (Qiagen) 113 genes panel.<br><br>DNA was extracted using the AllPrep DNA kit (cat# 80224, QIAGEN). DNA quantity and quality was assessed using Agilent 4200 TapeStation (Agilent, USA) for DINe (DNA Integrity Number equivalent). Libraries for whole exome sequencing (WES) were performed using Kapa Hyper Plus Library Prep Kits and the Agilent SureSelectXT V6 target

Confidential

	enrichment kit as described previously. Paired-end sequencing was performed using the NovaSeq 6000 S2 flow cell (2x100 cycles; Illumina).<br><br>FASTQ files were generated from the sequencer's output using Illumina bcl2fastq2 software (v.2.17.1.14, Illumina) with the default settings. All sequencing reads were aligned to the human genome reference sequence (GRCh37-hg19) using the BWA-MEM algorithm (v. 0.7.12). Picard tools (v.2.1.0) were used to remove PCR duplicates and to calculate sequencing metrics for quality control check. The Genome Analysis Toolkit (GATK, v. 3.5-0) was applied to realign local indels, recalibrate base scores, and identify genetic variants. Somatic point mutations and small indels were called using paired tumor-normal design using MuTect2 with stand_call_conf 30 and stand_emit_conf 30. Somatic variant was further filtered by quality PASS, coverage depth > 10 and allele frequency >5%. By comparing tumor DNA to its matched germline DNA control, copy number estimation was obtained through modified ASCAT2 package using 1) BAF data matrix derived from GATK variants calling and 2) LogR data matrix of sequencing coverage at GATK variant location from Picard CalculateHsMetrics.
**BRCA2, CDK12, PALB2 and TP53**	BRCA2, CDK12, PALB2 and TP53 pathogenic status was defined as samples with deleterious genomic alterations detected by NGS. Mutation calls from targeted NGS were reviewed manually using Integrative Genomics Viewer (14). Mutation annotation was based on available data from public databases (ClinVar, COSMIC, Human

C Confidential

	Genome Mutation Database, IARC TP53 Database), published literature, and in silico prediction tool.
**TMB**	Using the WES data, TMB was defined as the sum of somatic non-synonymous mutations/exome and was calculated using MuTect (version 1) and Variant Effect Predictor (15). The cutoff of 175 mutations/exome for TMB-high versus low as calculated from WES corresponds to the FDA approved clinical cutoff for TMB-high of 10 mutations per megabase (mut/mb) using the FoundationOne CDx (F1CDx) assay (16).
**mRNA-Seq**	Tumor RNA-Seq libraries were prepared according to the manufacturer's protocol using NEBNext® Ultra II Directional RNA Library Prep Kit for Illumina® NEB (#E7760) and ribo depletion using the NEBNext rRNA Depletion Kit (Human/Mouse/Rat) (NEB #6310). All sequencing was performed on the Illumina NextSeq 500 platform (Illumina) with 2 × 75bp read length. FASTQ files were generated using the BCL2FASTQ software.<br><br>Sequencing reads were aligned to (human GRCh37/hg19) using Tophat2 (v2.0.7). Gene expression, Fragments Per Kilobase of transcript per Million mapped reads (FPKM), was calculated using Cufflinks.
**mRNA signatures**	10 RNA expression signatures representative of key tumor biology and microenvironment elements were derived as previously described (17). RNA signatures included Angiogenesis, Hypoxia, Glycolysis, Proliferation, MYC, RAS, Granulocytic and Monocytic Myeloid-derived

	Suppressor cells (gMDSC, mMDSC respectively), Stroma/Epithelial to Mesenchymal Transition (EMT)/Transforming Growth Factor Beta (TGFβ), and WNT.

Supplementary Table 1 shows the assay methods for biomarkers of interest. Metastatic CRPC biopsies were assayed by WES, RNA seq, targeted NGS, Nanostring and IHC using previously described methods as described briefly above (8-17). A pathologist with prostate cancer expertise reviewed tissue blocks. The IHC slides were then digitized at high resolution (200x) using the ZEISS Axio Scan Z1 digital slide scanner (Carl Zeiss AG, Oberkochen, Germany). A pathologist-supervised machine learning algorithm (HALO AI, Indica Labs, New Mexico, USA) was trained to recognize prostate cancer cells and surrounding benign stroma. Color deconvolution for DAB and hematoxylin stains were performed. Cell recognition and nuclear segmentation was optimized for neoplastic cells. A visual threshold was set for positive staining. The analysis algorithm was adjusted to provide continuous data on the percentage of neoplastic cells staining positively, separately for each automatically annotated tumor region to facilitate distinguishing between staining in neoplastic cells and stromal positivity.

Supplementary Table 2. IHC assays methods for ATM, MLH1, PMS2, MSH2, MH6, PTEN and SOX2

Marker	Code / catalogue number	Supplier	Species	Staining platform	Retrieval buffer and method	Dilution and incubation time	Staining assessment
ATM	ab32420	Abcam	Rabbit monoclonal	BioGenex i6000	pH 9 Target Retrieval Solution Pressure cooker	1:400 1 hour	Nuclear histo-score

Confidential

MLH1	M3640	Dako	Mouse Monoclonal	BioGenex i6000	pH 8.1 Tris/EDTA Solution Pressure cooker	1:100<br>1 hour	Binary fashion between absent and present
PMS2	M3647	Dako	Rabbit monoclonal	BioGenex i6000	pH 8.1 Tris/EDTA Solution Pressure cooker	1:100<br>1 hour	Binary fashion between absent and present
MSH2	M3639	Dako	Mouse Monoclonal	BioGenex i6000	pH 8.1 Tris/EDTA Solution Pressure cooker	1:50<br>1 hour	Binary fashion between absent and present
MSH6	M3646	Dako	Rabbit monoclonal	BioGenex i6000	pH 8.1 Tris/EDTA Solution Pressure cooker	1:500<br>1 hour	Binary fashion between absent and present
PTEN	9559	Cell Signaling Technology	Rabbit monoclonal	BioGenex i6000	pH 6 Citrate with Tween Microwave	1:250<br>1 hour	Binary fashion between absent and present

SOX2	3579	Cell Signaling Technology	Rabbit monoclonal	Bond RX	pH 6 Epitope Retrieval 1	1:100<br>15 minutes	Nuclear histo-score

Supplementary Table 3. Clinical characteristics by selected biomarkers

	**Overall population (n = 100)**	**PD-L1 (n=23)**	**GEP (n=24)**	**TMB (n=12)**	**SOX2 (n=27)**	**NeuFea (n=9)**	**dMMR (n=7)**	**ATM loss (n=14)**	**BRCA2 (n=11)**	**CDK12 (n=4)**	**PALB2 (n=1)**	**PTEN loss (n=31)**	**p53 (n=25)**
ECOG at index date													
0	13 (13.3)	1 (4.5)	4 (17.4)	1 (9.1)	4 (14.8)	1 (11.1)	1 (14.3)	2 (14.3)	1 (10.0)	1 (25.0)	0	3 (10.0)	1 (4.0)
1	79 (80.6)	20(90.9)	18(78.3)	10(90.9)	23(85.2)	8 (88.9)	5 (71.4)	11(78.6)	8 (80.0)	3 (75.0)	1 (100.0)	25(83.3)	22(88.0)
≥2	6 (6.1)	1 (4.5)	1 (4.3)	0	0	0	1 (14.3)	1 (7.1)	1 (10.0)	0	0	2 (6.7)	2 (8.0)
Missing	2	1	1	0	0	0	0	0	1	0	0	1	0
Age at index date in years (IQR)	68.8 (64.8; 73.0)	67.0 (63.5; 72.5)	68.5 (65.5; 72.0)	67.0 (64.5; 70.5)	68.0 (64.0; 74.5)	58.0 (54.0; 71.0)	70.0 (64.5; 73.5)	68.0 (65.0; 73.8)	66.0 (61.5; 70.5)	67.0 (63.8; 69.2)	69.0 (69.0, 69.0)	67.0 (64.5; 73.5)	71 (63.0; 74.0)
Gleason score at diagnosis >7	64 (68.8)	6 (69.6)	18 (85.7)	7 (63.6)	19 (76.0)	6 (85.7)	5 (71.4)	57 (70.4)	8 (80.0)	4 (100.0)	1 (100.0)	18 (60.0)	19 (79.2)
Missing	7	0	3	1	2	2	0	2	1	0	0	1	1
T stage at diagnosis													
1-2	12 (14.0)	2 (9.0)	1 (4.8)	4 (40.0)	3 (14.3)	0	2 (28.6)	2 (14.4)	3 (30.0)	0	0	2 (7.6)	0
3-4	59 (68.6)	17(77.3)	15(71.5)	5 (50.0)	16(76.2)	5 (83.3)	4 (57.2)	7 (53.9)	6 (60.0)	2 (50.0)	1 (100.0)	23(88.5)	18(81.8)
X	15 (17.4)	3(13.69)	5 (23.8)	1 (10.0)	2 (9.5)	1 (16.7)	1 (14.3)	4 (30.8)	1 (10.0)	2 (50.0)	0	1 (3.8)	4 (18.2)
Missing	14	1	3	2	6	3	0	1	1	0	0	5	3

Confidential

N stage at diagnosis													
0	29 (35.8)	8 (36.4)	7 (36.8)	5 (45.5)	7 (31.8)	1 (14.3)	3 (42.9)	3 (25.0)	5 (45.5)	1 (33.3)	0	15(57.7)	6(28.6)
1	35 (43.2)	11(50.0)	8 (42.1)	4 (36.4)	11(50.0)	4 (57.2)	2 (28.6)	7 (58.3)	4 (36.4)	1 (33.3)	1 (100.0)	8 (30.8)	11(52.4)
X	17 (21.0)	3(13.69)	4 (21.1)	2 (18.2)	4 (18.2)	2 (28.6)	2 (28.6)	2 (16.7)	2 (18.2)	1 (33.3)	0	3 (11.5)	4 (19.0)
Missing	19	1	5	1	5	2	0	2	0	1	0	5	4
M stage at diagnosis													
0	35 (41.7)	7 (31.8)	6 (31.6)	6 (50.0)	8 (38.1)	3 (42.9)	4 (57.1)	5 (35.7)	7 (63.6)	0	1 (100.0)	13(50.0)	7 (31.8)
1	46 (54.8)	14(63.6)	13(68.4)	5 (41.7)	12(57.1)	4 (57.1)	3 (42.9)	9 (64.3)	4 (36.4)	2(100.0)	0	12(46.2)	14(63.6)
X	3 (3.6)	1 (4.5)	0	1 (8.3)	1 (4.8)	0	0	0	0	0	0	1 (3.8)	1 (4.5)
Missing	16	1	5	0	6	2	0	0	0	2	0	5	3

Supplementary table 3 shows clinical characteristics of the target population by selected biomarkers. Of note, biomarkers are not mutually exclusive, and this affects sample size for clinical variables.

C Confidential

Supplementary Table 4. Percentages of biopsy sites for PD-L1 and TcellinfGEP

	PD-L1 neg. (n=47)	PD-L1 pos. (n=23)	GEP neg. (n=69)	GEP pos. (n=24)
bone	0 (0%)	0 (0%)	19 (27.5%)	10 (41.7%)
nodal	34 (72.3%)	20 (87%)	36 (52.2%)	12 (50%)
other	1 (2.1%)	0 (0%)	1 (1.4%)	0 (0%)
soft tissue	8 (17%)	1 (4.3%)	8 (11.6%)	1 (4.2%)
visceral	4 (8.5%)	2 (8.7%)	5 (7.2%)	1 (4.2%)

Supplementary Table 4 shows the percentages of biopsy sites for PD-L1 and TcellinfGEP. Among samples that were negative for PD-L1 expression (n=47), 8 of these biopsies were from soft tissue (17%), whereas in samples that were positive for PD-L1 expression (n=23), 1 was from soft tissue (4.3%). For samples positive for TcellinfGEP expression (n=69), 19 were from bone (27.5%) and 8 from soft tissue biopsies (11.6%). This is in contrast to samples that were positive for TcellinfGEP expression (n=24), where 10 were from bone biopsies (41.7%) and 1 was from soft tissue (4.2%).

Supplementary Table 5. Correlations of dMMR, high TMB, PD-L1 and mutation origin (germline/ somatic) within the target population.

Patient ID	MSH2 deleteriouss alteration (Yes/No)	Subtype of deleterious alteration	MSH2 IHC loss (Yes/No)	MSH6 Deleterious	Subtype of deleterious alteration	MSH6 IHC loss (Yes/No)	Germline mutation (Yes/No)	Bi-allelic	High TMB (Yes/No)	PD-L1 expression (Yes/No)

Confidential

				alteration (Yes/No)				hit (Yes/No)		
059	No	N/A	Yes	Yes	p.I225fs*22	Yes	Yes	Yes	Yes	No
060	Yes	Deep deletion	Yes	No	N/A	Yes	No	Yes	Yes	Yes
062	Yes	Deep deletion	Yes	Yes	Deep deletion	Yes	No	Yes	Yes	No
010	No	N/A	Yes	No	N/A	Yes	No	No	Yes	No
032	No	N/A	No	Yes	p.T128Nfs*	No	Yes	No	No	No
035	No	N/A	No	Yes	p.S580L	No	Yes	No	No	No
039	No	N/A	Yes	No*	N/A	Yes	No	Yes*	Yes	No

C Confidential

C Confidential

Supplementary Figures 1a-h. Kaplan Meier plots showing median OS for BRCA2, TMB, NeuFeat, dMMR, ATM, CDK12, PTEN and p53 at 36 months

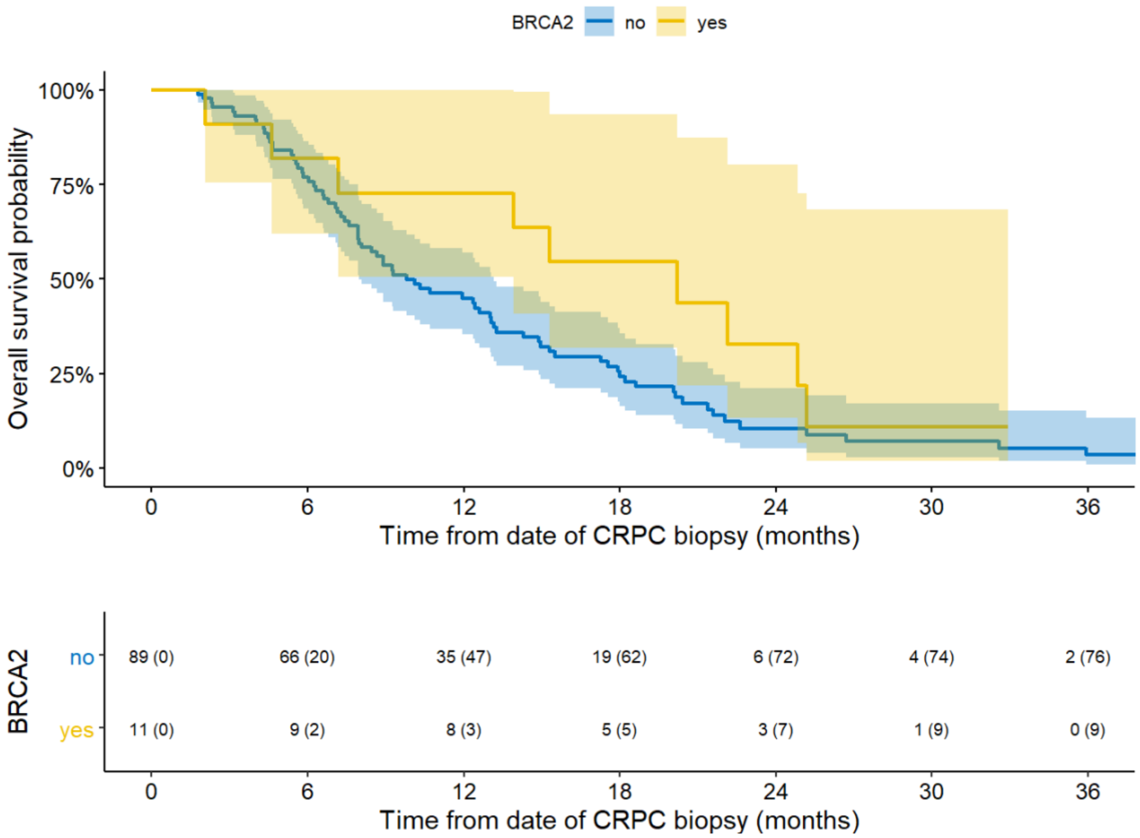

Supplementary figure 1a shows KM curves for median OS censored at 36 months for patients with (yellow line) and without (blue line) BRCA2 deleterious alterations. Patients with BRCA2 deleterious alterations showed a trend to better OS (20 vs 10 months).

Confidential

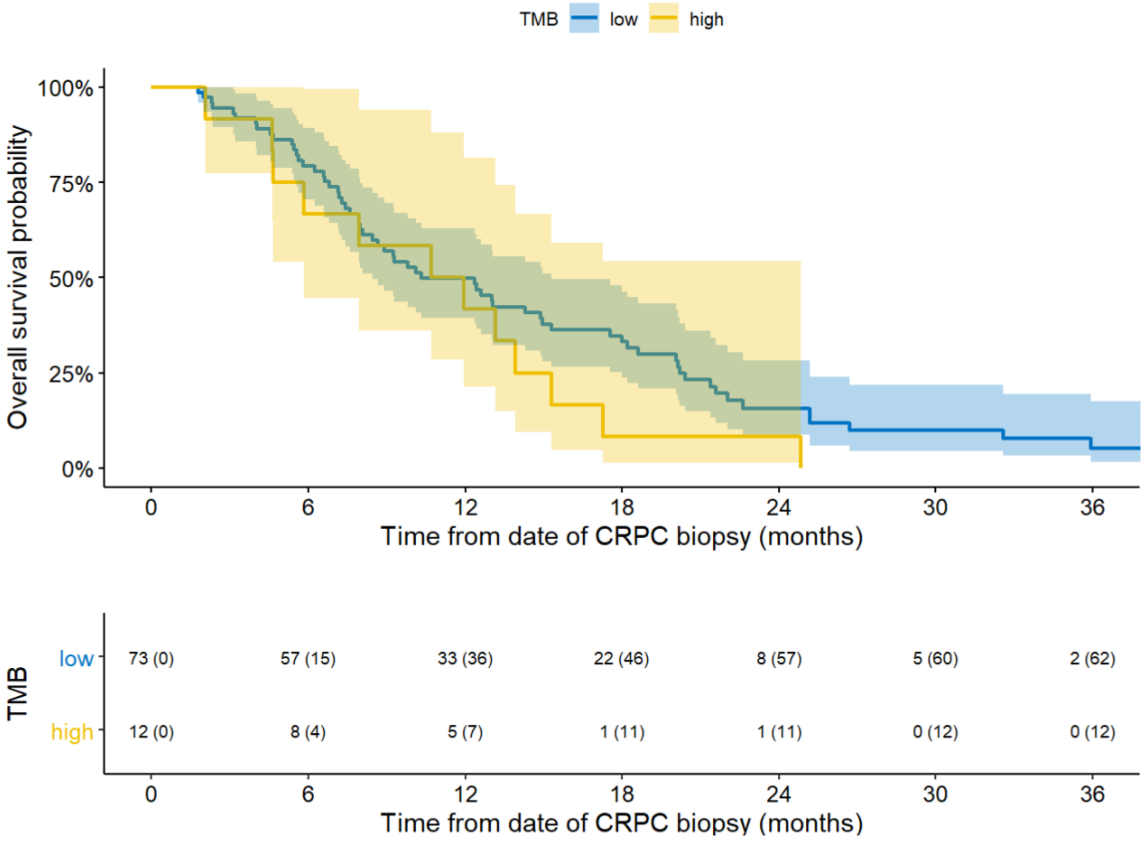

Supplementary figure 1b shows median OS censored at 36 months for patients with high vs low TMB (10 vs 11 months).

Confidential

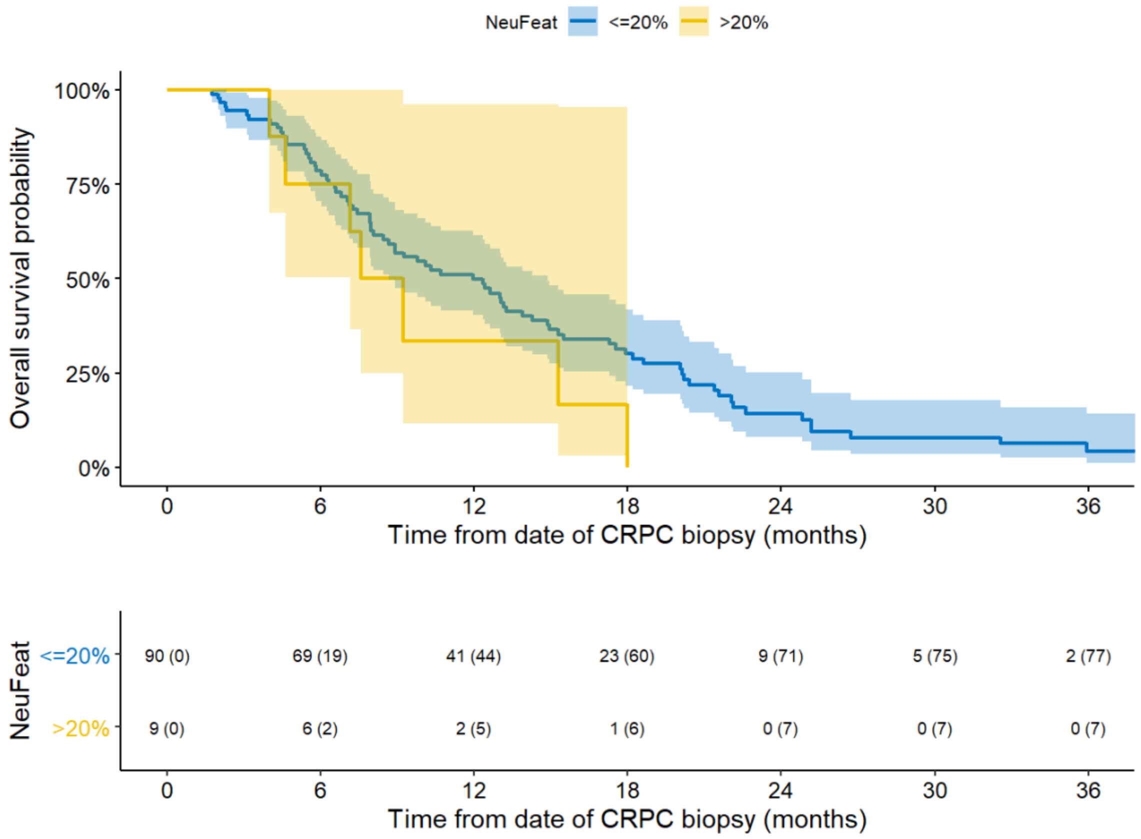

Supplementary figure 1c shows KM curves for median OS censored at 36 months for patients with (yellow line) and without (blue line) neuroendocrine features in the mCRPC biopsy (8 vs 12 months).

Confidential

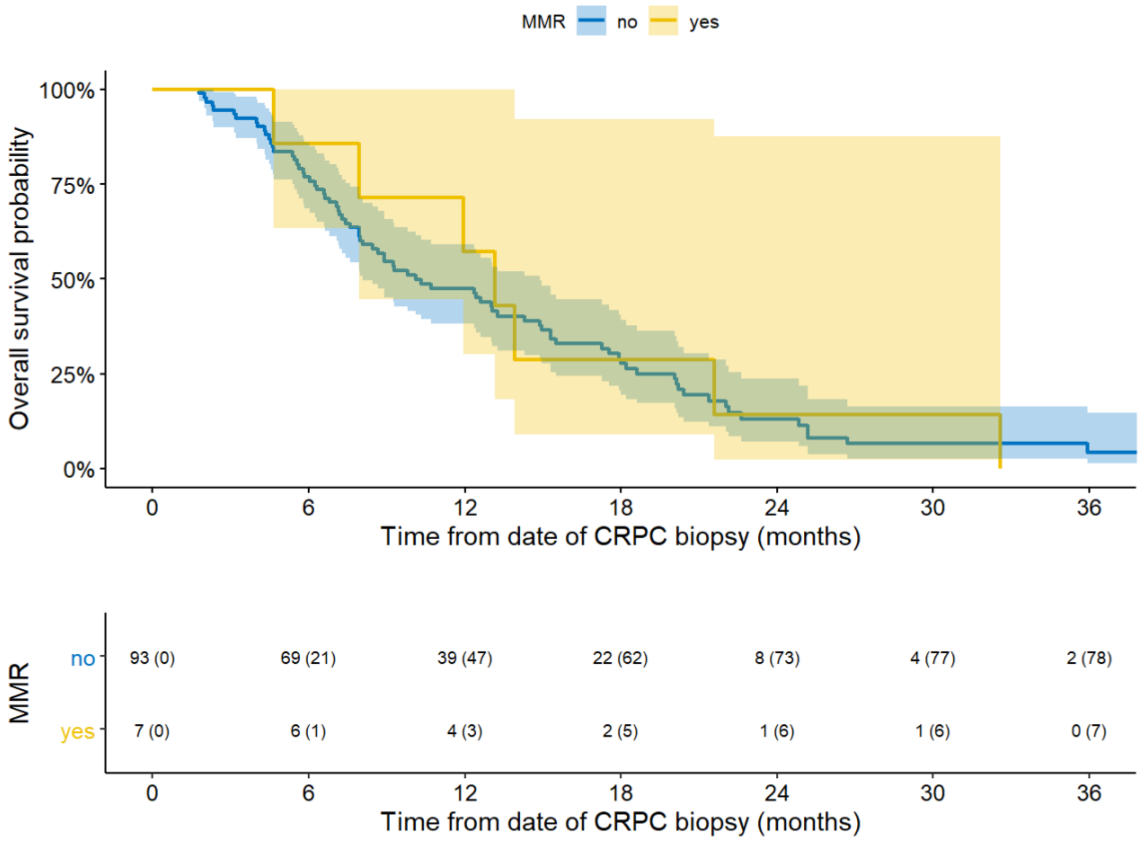

Supplementary figure 1d shows KM curves for median OS censored at 36 months for patients with (yellow line) and without (blue line) MMR loss (13 vs 10 months).

Confidential

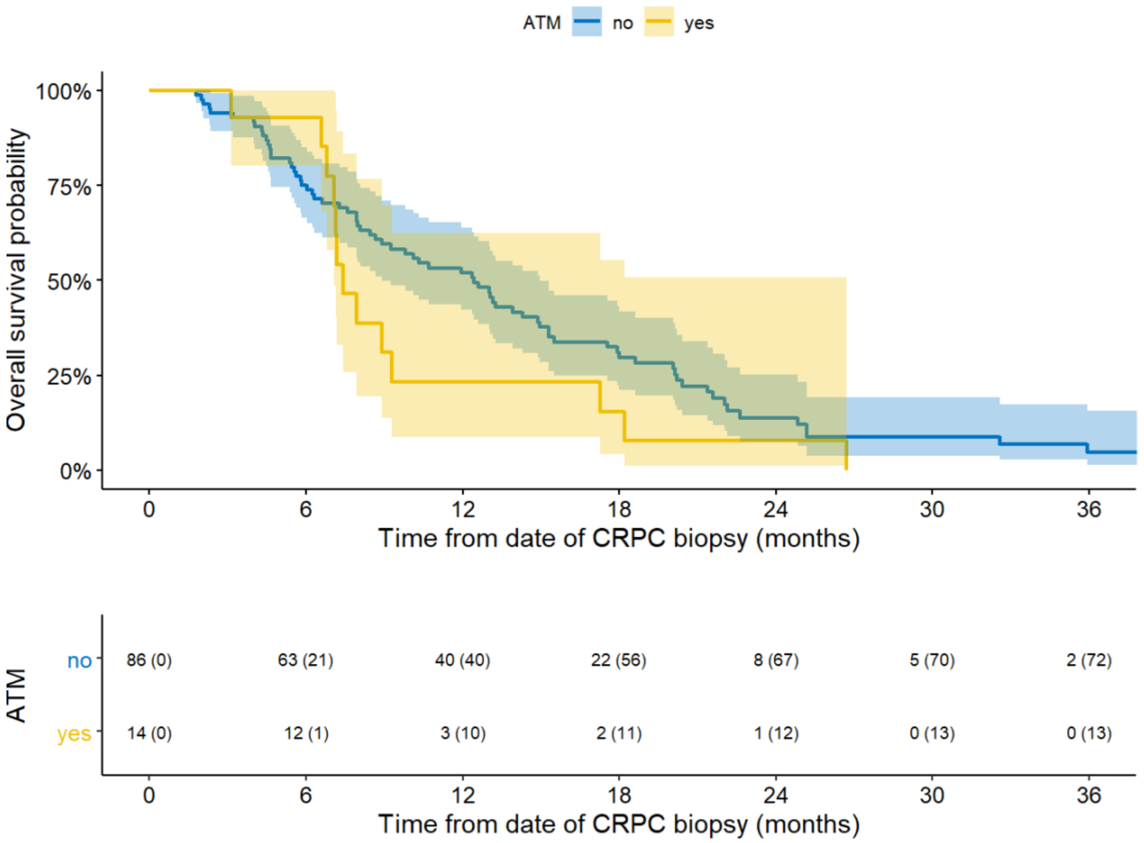

Supplementary figure 1e shows KM curves for median OS censored at 36 months for patients with (yellow line) and without (blue line) ATM loss (7 vs 12 months).

Confidential

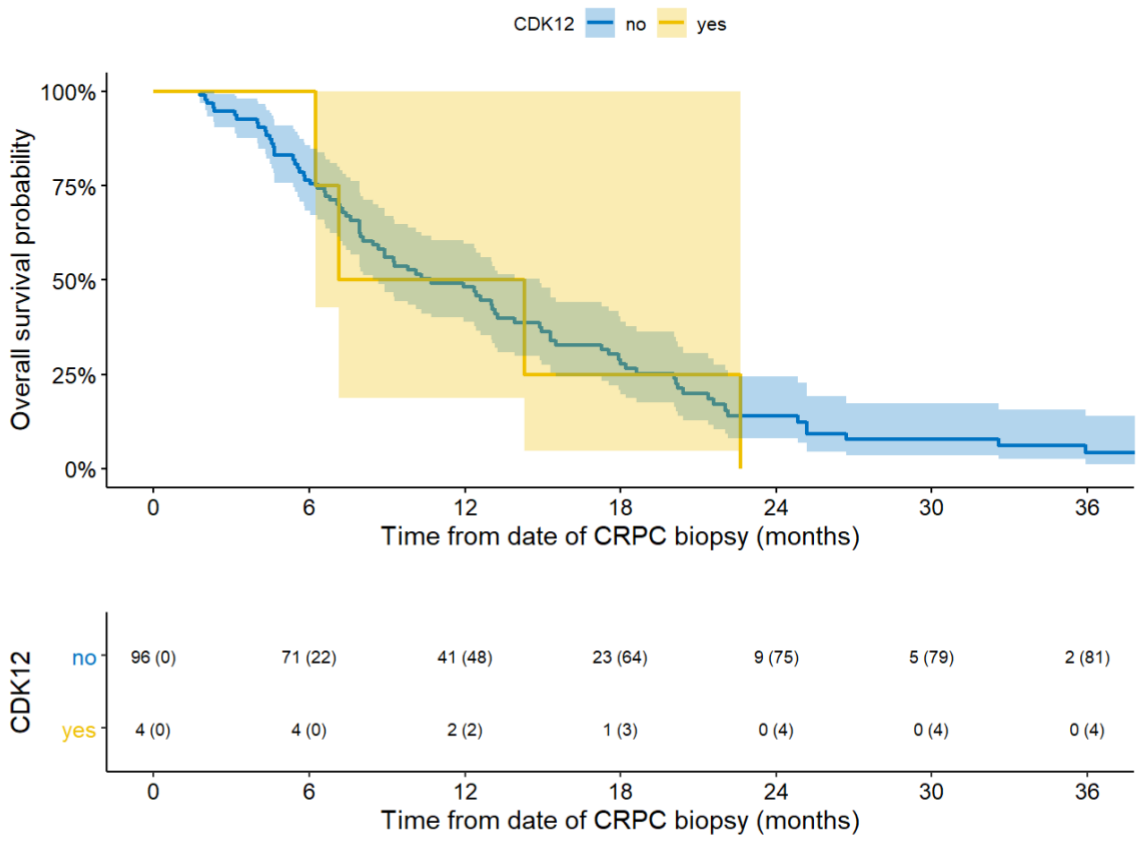

Supplementary figure 1f shows KM curves for median OS censored at 36 months for patients with (yellow line) and without (blue line) CDK2 deleterious genomic aberrations (11 months for both subgroups).

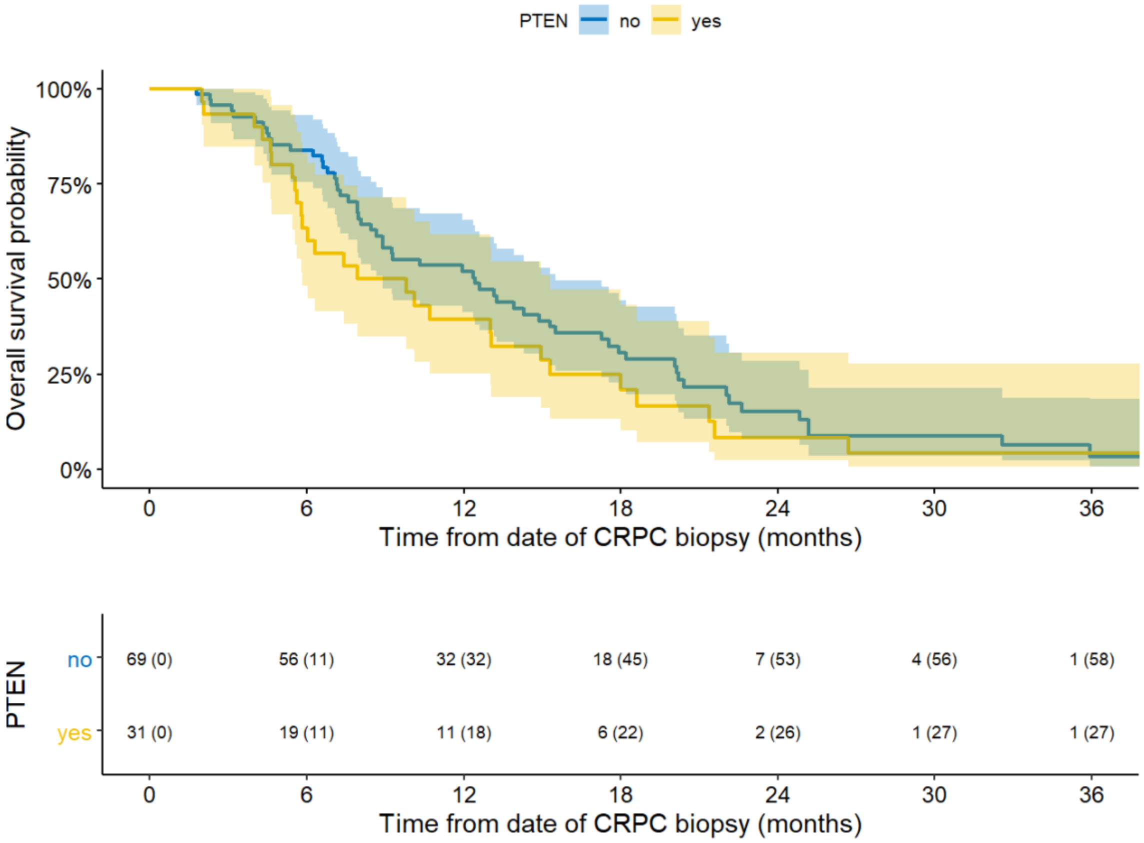

Supplementary figure 1g shows KM curves for median OS censored at 36 months for patients with (yellow line) and without (blue line) PTEN loss (9 vs 12 months).

C Confidential

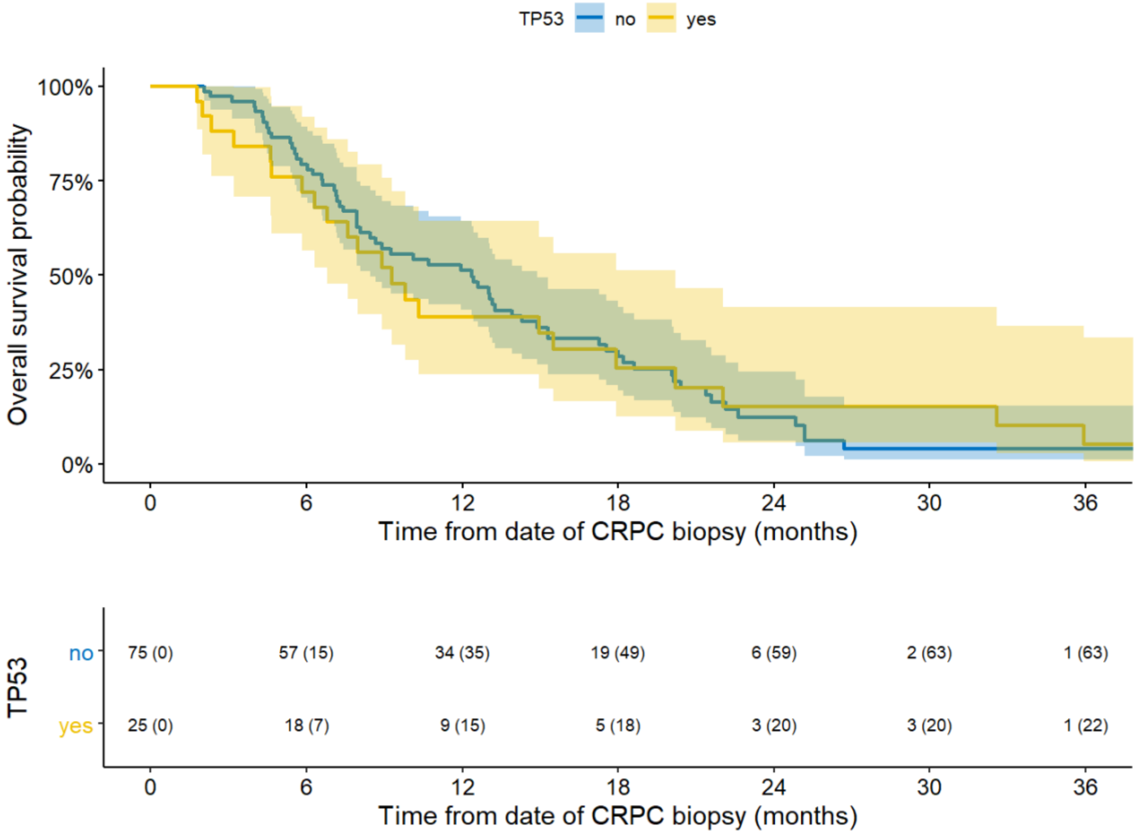

Supplementary figure 1h shows KM curves for median OS censored at 36 months for patients with (yellow line) and without (blue line) TP53 deleterious aberrations (9 vs 12 months).

Confidential

Supplementary Figure 2. Adjusted Hazard ratios for association of mRNA signatures (per SD increase*) and overall survival. Adjusted model controlling for age at CRPC biopsy, ECOG, log-transformed PSA, Gleason Score, and liver metastases.

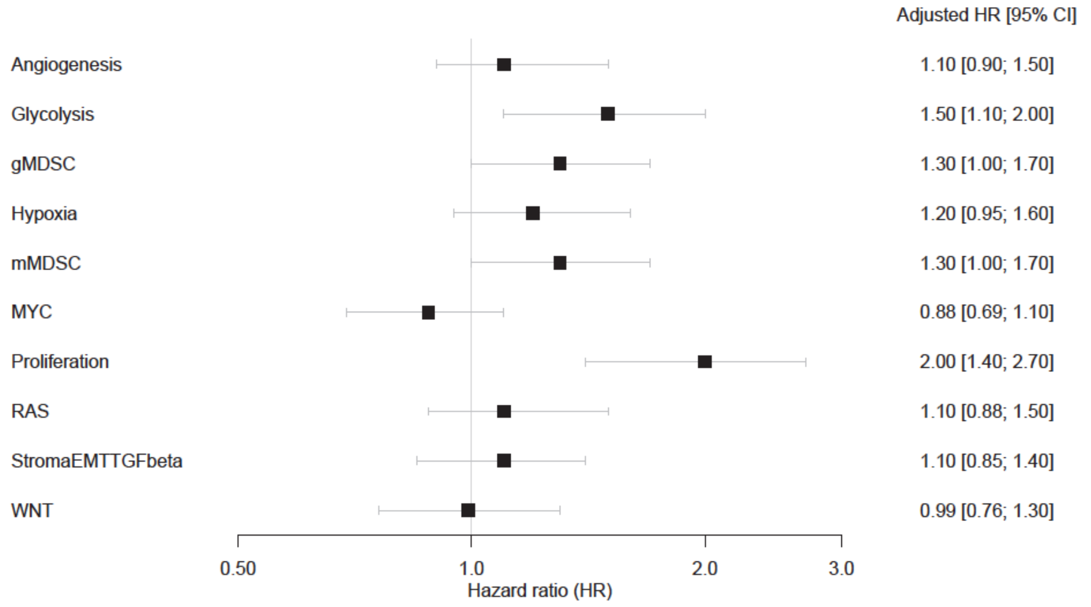

gMDSC=granulocytic myeloid-derived suppressor cells
mMDSC=monocytic myeloid-derived suppressor cells

* Before applying Cox regression models, each of 10 mRNA expression signatures was standardized separately by subtracting the mean and dividing by the standard deviation to shift the distribution to have a mean of zero and a standard deviation of one

C Confidential

Supplementary Figure 3. Correlations between mRNA signatures and biomarkers of interest.

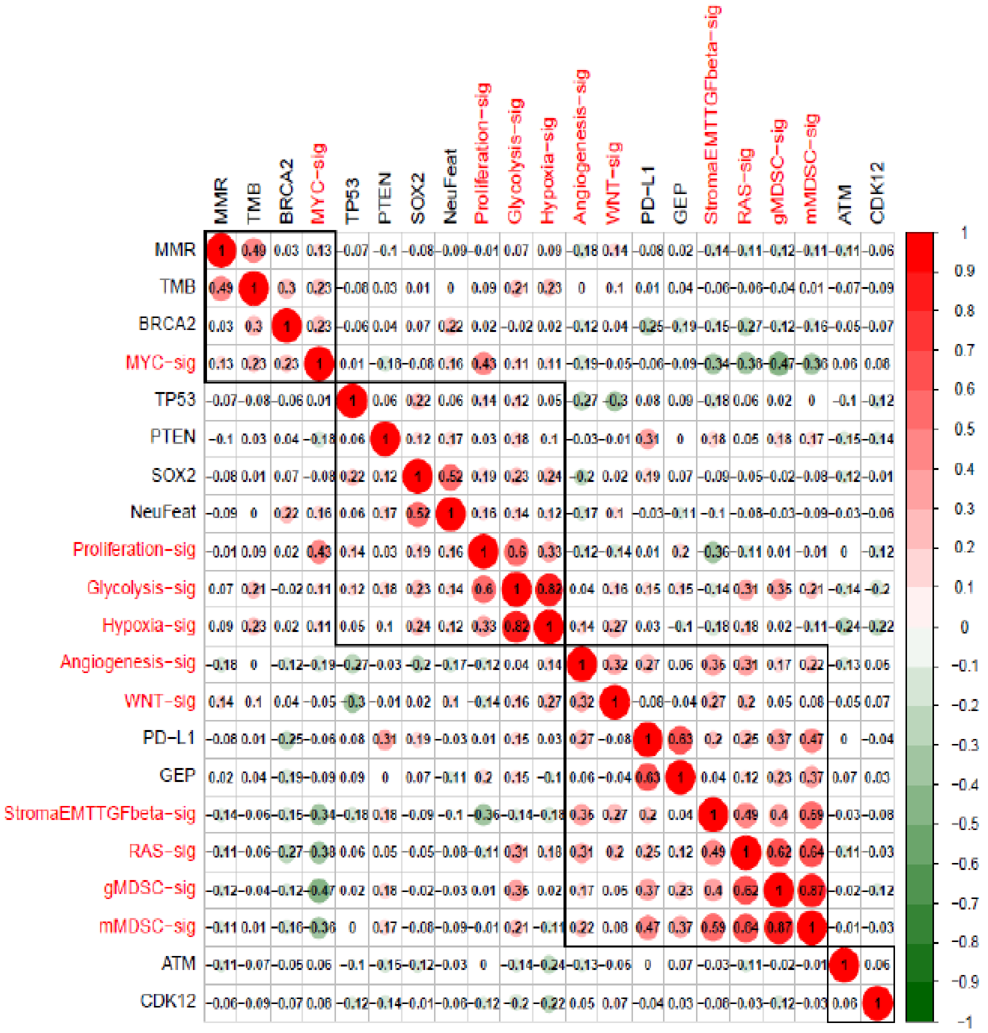

Supplement: Supplementary Material [file EMS207309-supplement-Supplementary_Material.pdf]
