# Supplementary material for: Immune Biomarkers in Metastatic Castration-resistant Prostate Cancer
Source: Eur Urol Oncol. Author manuscript; Available in PMC 2025 Aug 8. (PMC7617991; doi:10.1016/j.euo.2022.04.004)
Supplement: Supplementary Appendix [file EMS207309-supplement-Supplementary_Appendix.pdf]

Appendix A. Supplementary data

Supplementary data to this article can be found online at https://doi.org/10.1016/j.euo.2022.04.004.
